# Enhancing Nonenzymatic Glucose Detection Through Cobalt‐Substituted Hafnia

**DOI:** 10.1002/advs.202408687

**Published:** 2025-02-24

**Authors:** Jeonghyeon Oh, Avis Sin Hui Wee, Eun‐Byeol Park, Jaejin Hwang, Seon Je Kim, Hu Young Jeong, Myat Thet Khine, Pavan Pujar, Jaekwang Lee, Young‐Min Kim, Sunkook Kim

**Affiliations:** ^1^ Multifunctional Nano Bio Electronics Lab School of Advanced Materials Science and Engineering Sungkyunkwan University Suwon Gyeonggi‐do 16419 Republic of Korea; ^2^ Department of Energy Science Sungkyunkwan University (SKKU) Suwon Gyeonggi‐do 16419 Republic of Korea; ^3^ Department of Physics Pusan National University Busan 46241 Republic of Korea; ^4^ Graduate School of Semiconductor Materials and Devices Engineering Ulsan National Institute of Science and Technology (UNIST) Ulsan 44919 Republic of Korea; ^5^ Department of Ceramic Engineering Indian Institute of Technology (IIT‐BHU) Varanasi Uttar Pradesh 221005 India

**Keywords:** chemical solution deposition, nonenzymatic glucose sensing, oxygen vacancy

## Abstract

Engineered defect chemistry in ultrathin (≈5 nm) hafnia through substitutional cobalt (HCO) is investigated for selective glucose sensing. Thin films of HCO, grown using chemical solution deposition (CSD)—traditionally used to grow thick films—on silicon, show significant glucose sensing activity and undergo monoclinic to orthorhombic phase transformation. The presence of multivalent cobalt in hafnia, with oxygen vacancies in proximity, selectively oxidizes glucose with minimal interference from ascorbic acid, dopamine, and uric acid. Theoretical investigations reveal that these oxygen vacancies create a shallow donor level that significantly enhances electrocatalytic activity by promoting charge transfer to the conduction band. This results in considerable selectivity, repeatability, and reproducibility in sensing characteristics. These findings highlight the technological importance of using CSD for thin films, paving the way for ultrathin CSD‐processed HCOs as potential candidates for selective glucose sensing applications.

## Graphical abstract

Proposed nonenzymatic glucose sensor utilizes ultrathin hafnia films engineered with cobalt and oxygen vacancies, grown via chemical solution deposition. Cobalt atoms with multivalent state act as sites of oxidation for glucose and oxygen vacancies enhance charger transfer, allowing precise glucose oxidation with minimal interference from other biomolecules. The chemical solution deposition‐hafina technology confirms its suitability for high‐quality nonenzymatic glucose sensor.

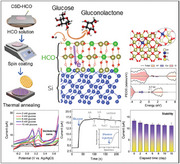

## 1 Introduction

Diabetes mellitus, a chronic disease in which a patient has a metabolic disorder in the endocrine system with abnormally high blood glucose levels, is responsible for one death in every fifth second in 2021.^[^ ^1^ ^]^ It is one of the major noncommunicable and growing diseases throughout the world, increased by 3% over the last 20 years, and estimated that more than 650 million adults develop diabetes by the year 2045.^[^ ^2^ ^]^ Diabetic patients have problems producing (Type 1) or efficiently using (Type 2) insulin hormone in the body. In both cases, patients experience abrupt changes in glucose concentration in the blood. If glucose in the blood is not controlled at appropriate levels, it can chronically result in severe complications, such as cardiovascular disease, retinopathy, neuropathy, and diabetic kidney disease.^[^ ^3^ ^]^ Clinical treatments for diabetes are mainly focused on the management of glycemic control for near‐normal glucose levels, achieved by continuous glucose monitoring (CGM) systems. Therefore, the CGM device should be designed to detect glucose with high accuracy, fast response, excellent selectivity, and long‐term stability. The CGM device mainly constitutes the glucose sensor, a transmitter, and a receiver; the important part of the above is the sensor. It utilizes various methods of sensing via optical, transdermal, thermal, and electrochemical routes.^[^ ^4^ ^]^

Amidst these, a promising technique is the electrochemical analysis method, owing to its stability, implantability, portable easiness, and comparatively lower in cost to precisely determine the glucose levels.^[^ ^5^ ^]^ The electrochemical glucose sensors are based on both enzymatic and nonenzymatic, the former uses glucose oxidase (GO*_x_*), and the latter is based on inorganic artificial enzymes. Commercial CGM devices such as Abbott, Dexcom, and Medtronic sensors have developed electrochemical detection technology for glucose based on glucose oxidase.^[^ ^6^ ^]^

However, glucose detection using enzymes is greatly limited by the following factors: poor stability due to biofouling changes in enzyme catalytic activity due to pH, temperature, humidity, and toxic chemicals and the need for additional redox mediators such as ferrocene derivatives, ferricyanide, and so on.^[^ ^7^ ^]^ Redox mediators are introduced to address issues such as oxygen dependence and slower rate of electron transfer of glucose biosensors using only GO*_x_*.^[^ ^8^ ^]^ However, the immobilization of redox mediators and enzymes onto the electrode surface for long‐term use is challenging, necessitating the development of glucose sensors capable of direct electron transfer (DET) from glucose to the electrode surface without the need for redox mediators.^[^ ^9^ ^]^

These drawbacks account for the exploration of nonenzymatic glucose sensing materials with promising electrochemical activity, biocompatibility, and facile fabrication.^[^ ^10^ ^]^ Thus far, pure metals and their metal alloys,^[^ ^11^ ^]^ carbon‐based composites,^[^ ^12^ ^]^ and functional metal oxides^[^ ^13^ ^]^ have been widely investigated for their nonenzymatic glucose sensing activity. Taking noble metal as an example, gold (Au) and platinum (Pt) are widely investigated due to their sufficient stability and excellent electrochemical performance. However, their high‐cost leads to the construction of glucose nonenzyme sensors by metals abundant in nature such as transition metal (Ni, Cu, and Co) and Zn, Fe, Mo, etc.^[^ ^14^ ^]^

Electrical conductivity of material is very important for electrochemical glucose detection hence it enables effective DET from glucose to the electrode surface, especially for metal oxides with poor conductivity. Carbon composite, conductive metal–organic frameworks (MOFs), or oxygen‐vacancy formation of metal oxides are often used to overcome their poorly conductive nature.^[^ ^15^ ^]^ The electrocatalytic activity of functional metal oxides is the outcome of inherent oxygen vacancies in them.^[^ ^16^ ^]^ Previous investigation on Co_3_O_4_ nanosheets with oxygen vacancies has proven beneficial not only for larger surface areas but also for enhanced electrical conductivity due to oxygen vacancies;^[^ ^17^ ^]^ other studies include, various functional metal oxides, such as CuO, and HfO_2_.^[^ ^18^ ^]^ Such characteristics of cobalt lead to investigate it as immunosensor and energy storage.^[^ ^19^ ^]^

Pristine HfO_2_ is a high dielectric permittivity oxide ceramic with low oxygen vacancy concentration.^[^ ^20^ ^]^ Due to this, it has fewer free electrons available for electrical conduction.^[^ ^21^ ^]^ While hafnium (Hf) exists primarily in the 4+ oxidation state, cobalt (Co) can have multiple oxidation states. Therefore, cobalt oxide can inherently contain vacancies arising from its multivalent characteristics, making it more effective for catalytic oxidation activities compared to HfO_2_.^[^ ^22^ ^]^ Therefore, a doped composition of hafnium oxide and cobalt oxide could serve as an active material, maintaining oxygen vacancies while reducing toxicity, given that pure cobalt oxide nanoparticles are known to be toxic and harmful to human health.^[^ ^23^ ^]^ We investigate the effects of oxygen vacancies in Co‐doped HfO_2_ (HCO) thin films directly grown on silicon and evaluate their efficacy in selective glucose sensing applications.

## 2 Results and Discussion

**Figure 1:**
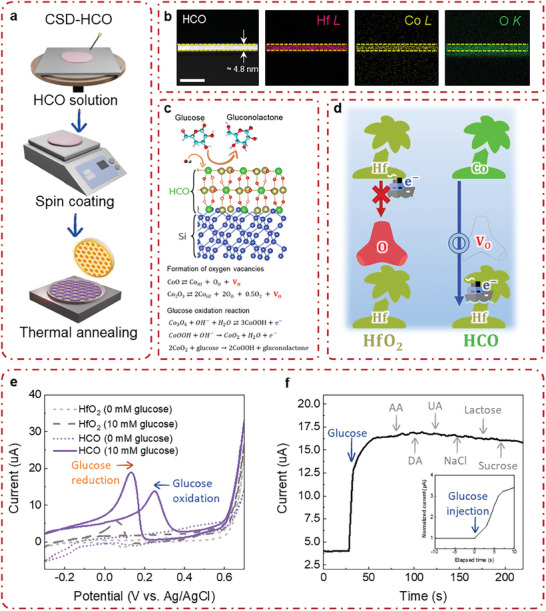
Conceptualization, fabrication, and application of CSD‐HCO electrode in nonenzymatic glucose sensing. a) Fabrication of HCO thin films using chemical solution deposition (CSD) followed by rapid thermal annealing process. b) Nanoscale elemental distribution mapping of HCO thin film directly grown on silicon. c) Schematic illustration of HCO‐based nonenzymatic glucose sensing mechanism in 0.1 m NaOH, oxygen vacancy defect formation, and glucose oxidation reaction. d) Analogy of charge transfer for HfO_2_ and HCO due to electric conductivity by oxygen vacancies. e) Cyclic voltammetry (CV) response of pristine HfO_2_ at 0 mm glucose (dashed line, light gray in color), HfO_2_ at 10 mm glucose (long‐dashed line, gray in color), HCO at 0 mm glucose (dotted line, light purple in color), and HCO at 10 mm glucose (solid line, purple in color) in 0.1 m NaOH at the scan rate of 10 mV s^−1^. f) Chronoamperometric response of HCO upon successive addition of 10 mm glucose, 0.1 mm ascorbic acid (AA), 0.1 mm dopamine (DA), 0.1 mm uric acid (UA), 0.1 mm NaCl, 0.1 mm lactose, and 0.1 mm sucrose in 0.1 m NaOH at the peak of glucose oxidation (+0.25 V vs Ag/AgCl).

**Figure** **1**a represents the strategy adopted to deposit ultrathin Co‐doped HfO_2_ films using aqueous precursor‐based chemical solution deposition (CSD) method.^[^ ^24^ ^]^ The deposition involves two steps: first, the formation of an amorphous HCO thin film, followed by crystallization via rapid thermal annealing. Figure 1b depicts the thickness of HCO film (Co concentration ≈15%) to be ≈5 nm (Figure S1, Supporting Information), and the energy dispersive X‐ray spectroscopy (EDX) mapping of cross‐sectional HCO reveals the uniform distribution of Hf, Co, and O elements throughout the film thickness. The schematic in Figure 1c,d demonstrates the nonenzymatic glucose sensing mechanism of HCO under alkaline conditions. The formation of oxygen vacancies following Co substitution is demonstrated through defect reactions using Kröger–Vink notations. Based on the difference in relative binding energies between Co‐O and Hf‐O, oxygen vacancies are anticipated to form in close proximity to Co. These vacancies play a pivotal role in facilitating electron transfer between glucose molecules and the electrode surface, thereby enhancing the electrocatalytic performance of the glucose sensor, similar to traditionally investigated pristine cobalt oxide glucose sensors.^[^ ^13^, ^25^ ^]^

The glucose sensing activity of HfO_2_ with Co substitution (that is, HCO) is compared with pristine HfO_2_ in Figure 1e. The distinct peaks corresponding to oxidation and reduction indicate the impact of altered defect chemistry due to Co occupying the Hf site. Moreover, these oxidation and reduction reactions are exclusively attributed to blood glucose. Selectivity tests were conducted to assess the response of HCO to other interference species present in blood such as ascorbic acid (AA), dopamine (DA), uric acid (UA), sodium chloride (NaCl), lactose and sucrose. Such interference species can produce significant oxidation current even though their concentrations are less compared to glucose.^[^ ^26^ ^]^ Therefore, amperometric *i–t* responses with glucose and interference species were measured to investigate the anti‐interference ability of HCO electrode. Concentration of interference species was set to 0.1 mm, which is based on a normal physiological level.^[^ ^27^ ^]^ The results demonstrate exceptional sensitivity of HCO to glucose, with minimal change in output current observed for other interference species, as shown in Figure 1f.

**Figure 2:**
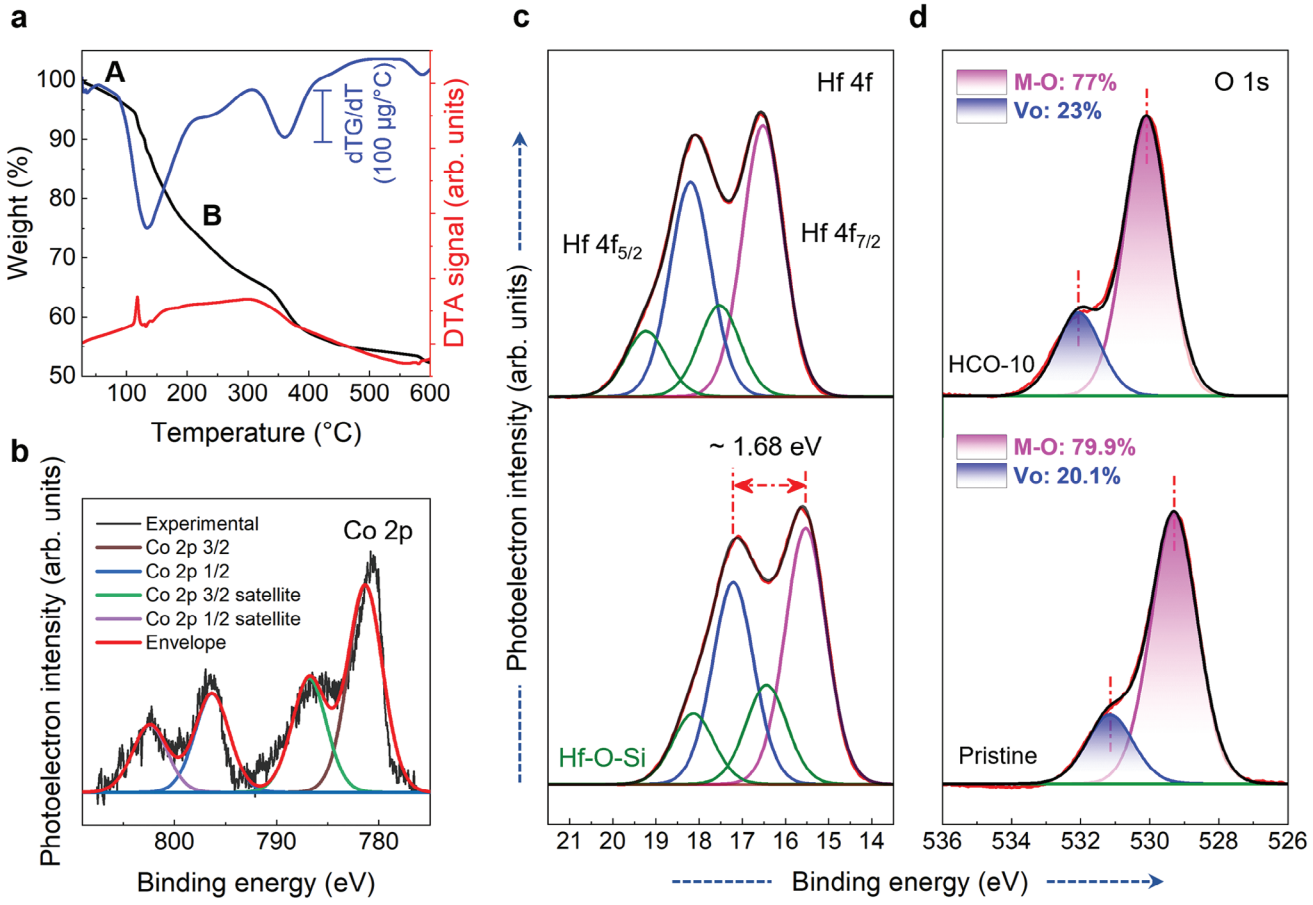
Thermal analysis of liquid‐phase precursor and surface chemical composition analysis of HCO thin films. a) TG/DTA trace of HCO precursor with the first derivative of TGA. Deconvolution of high‐resolution core level spectra of b) Co 2p, c) Hf 4f, and d) O 1s for both pristine HfO_2_ and HCO‐10; The presence of oxygen vacancies in HCO because of Co‐substitution is evident from the high‐resolution O 1s spectra.

The liquid‐phase precursor to HCO‐the desired metal oxide transformation temperature is predicted using thermal analysis of precursor. The liquid precursor of HCO is composed of Co(NO_3_)_2_ and HfCl_4_ as the sources of Co and Hf, respectively. The reaction between these chemical species yields a compositionally robust HCO. Along with these metal salts, the liquid phase precursor also contains a dissolving medium, which is a cosolvent of 2‐methoxyethanol (2‐ME) and deionized water. The thermal decomposition of liquid precursor is traced and presented in **Figure** **2**a. Two notable weight losses (at A and B) in the thermalgravimetric analysis (TGA) curve are designated to solvent evaporation and the decomposition of metal salts, respectively.^[^ ^28^ ^]^ After a temperature of 400 °C, the curve is nearly horizontal depicting no significant weight loss; this gives a clue to the formation of thermally stable oxide (i.e., HCO). These weight losses at A and B are also supported by the first derivative of the TGA curve (blue in color, Figure 2a). The prominent slope changes in TGA show peaks in the first derivative curve facilitating the effortless recognition of temperatures. Further, the corresponding differential thermal analysis (DTA) curve supports that the formation of HCO from precursor is continuous over a range of temperatures and has an onset at around 120 °C and is nearly accomplished at around 400 °C. Owing to these inputs, an annealing temperature of 350 °C is selected for the spin‐coated films.

The overall structure of the grown HCO films was examined by grazing incidence X‐ray diffraction (GIXRD) and compared with a pristine HfO_2_ counterpart (Figure S2, Supporting Information). Since the film grew to be as thin as ≈6 nm, broad diffraction signatures are observed, not allowing the determination of the exact crystal phase configuration. Nonetheless, the analysis of diffraction peak shape and position conveys useful information about structural geometry. To this end, the Scherrer equation was used to estimate the crystallite size of the CSD‐processed thin films.^[^ ^29^ ^]^

(1) $$D=\frac{K\lambda }{\beta \sin\theta }$$

where *D* is the crystallite size, *K* is the Scherrer constant (0.9), *λ* is the wavelength of the X‐ray source (0.15406 nm), *β* is the full width at half maximum (FWHM) in radians, and *θ* is the Bragg's diffraction angle. The crystallite sizes of pristine HfO_2_ and HCO samples range from ≈3 to 6 nm, which is expected for ultrathin films (Table S1, Supporting Information). As the Co doping concentration increases, the FWHM decreases, indicating an increase in crystallite size in the HCO samples. Moreover, the diffraction peaks shift toward higher 2θ values with increasing Co concentrations, which is attributed to the successful doping of Co ions into the HfO_2_ lattice. Since Co^2+^ (0.065 nm) and Co^3+^ (0.0545 nm) ions have smaller ionic radii than Hf^4+^ (0.071 nm) ions, doping leads to a shrinkage in the volume of the HfO_2_ unit cell. Consequently, this reduction in the lattice parameter causes an increase in Bragg's diffraction angle.^[^ ^30^ ^]^

X‐ray photoelectron spectroscopy (XPS) was further used to estimate the surface chemical composition of pristine HfO_2_ and HCO‐10 thin films (Figure 2b–d), with binding energies calibrated using C 1s peak centered at 284.6 eV (Figure S3, Supporting Information).^[^ ^31^ ^]^ The peak fitting parameters for each element are provided in Table S2a–d (Supporting Information). In Figure 2c, the core‐level spectrum of Hf 4f is deconvoluted into four peaks (two doublets) with a spin–orbit splitting of ≈1.68 eV. The two signature peaks at lower binding energies, positioned at 15.53 and 17.21 eV, correspond to the Hf 4f_7/2_ and Hf 4f_5/2_ (Hf*─*O bonding), respectively.^[^ ^32^ ^]^ On the other hand, the doublet at higher binding energies, centered at 16.44 and 18.13 eV, is assigned to Hf silicate (Hf*─*O*─*Si bonding).^[^ ^33^ ^]^ To gain further insights, the high‐resolution O 1s core spectrum is deconvoluted into two synthetic signatures at 530.1 and 532.1 eV, corresponding to lattice oxygen (i.e., metal–oxygen–metal) and nonlattice oxygen (i.e., oxygen‐deficient regions), respectively.^[^ ^34^ ^]^ The presence of oxygen vacancies was quantified from the area fraction of nonlattice oxygen. The relative fractions of oxygen vacancies in pristine HfO_2_ and HCO‐10 are 20.3% and 23.0%, respectively. Doping Co into the HfO_2_ crystal structure enhances the formation of oxygen vacancies, leading to improved electrochemical glucose sensing performance. Furthermore, the Co 2p core level spectrum depicts two Co 2p peaks at 781.3 and 796.3 eV, corresponding to Co 2p_3/2_ and Co 2p_1/2_ respectively, confirming the presence of Co^2+^ and Co^3+^ ions in HCO‐10 sample (Figure 2b).

The shift in core‐level binding energy due to the doping effect has been reported in several publications. Doping with an element of lower electronegativity can alter the interaction between atoms, resulting in a shift to lower binding energy, and vice versa.^[^ ^35^ ^]^ As shown in Figure 2c,d, both the Hf 4f and O 1s core levels shift to higher binding energies with Co doping. This shift is attributed to the incorporation of the higher electronegativity Co dopant (1.88) into the lower electronegativity Hf lattice (1.30).

**Figure 3:**
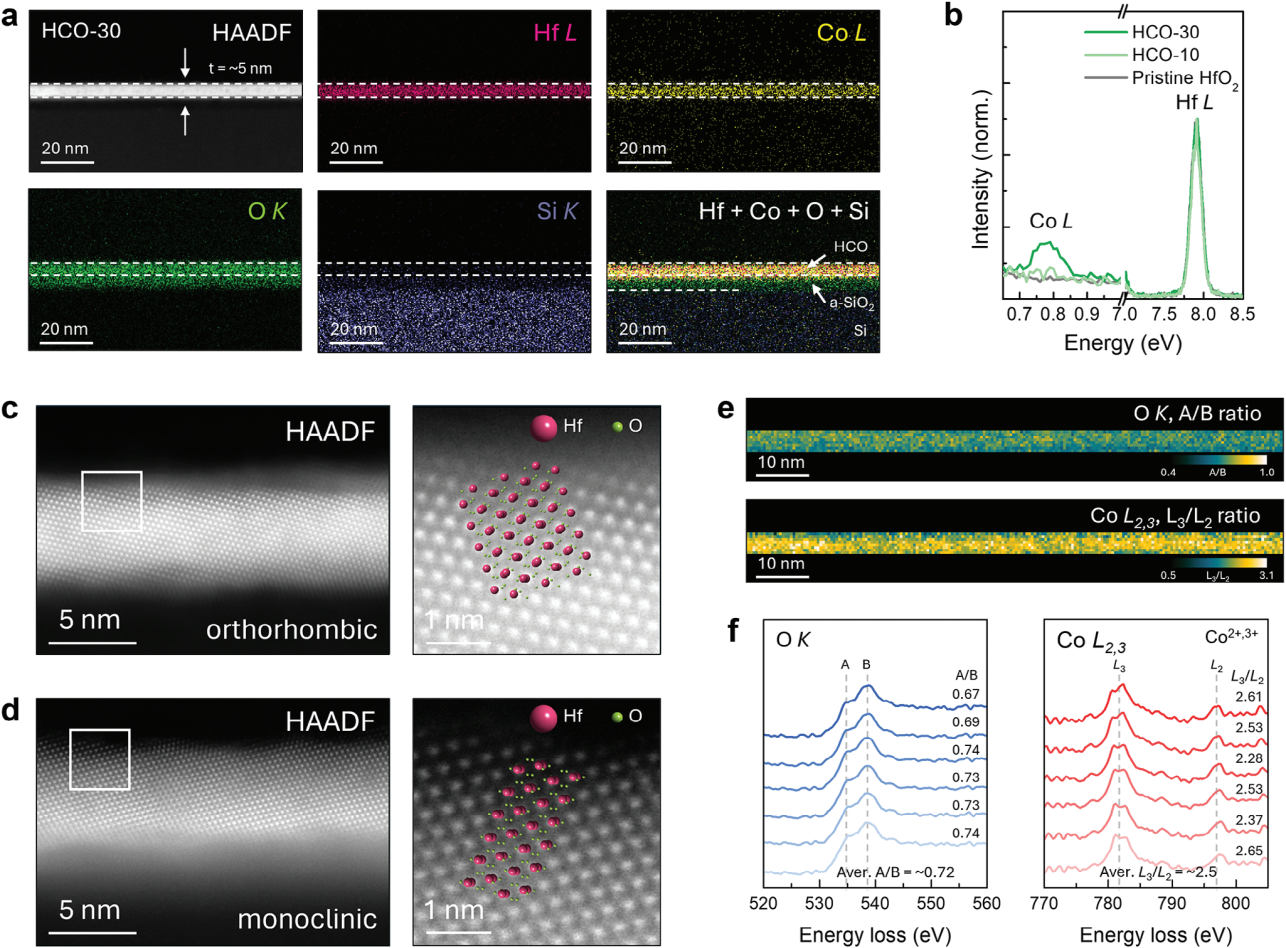
Elemental distribution, crystal phase, and chemical analyses. a) EDX elemental maps of Hf L (7.898 keV, pink), Co L (6.924 keV, yellow), O K (0.525 keV, green), and Si K (1.739 keV, purple) peaks for the HCO sample. b) Comparison of Co and Hf peaks for the three samples: undoped HfO_2_, HCO‐10, and HCO‐30, respectively. c,d) Representative high‐resolution STEM images of orthorhombic and monoclinic structures observed in HCO samples. e) Maps of (top) A/B ratio in O K edge and (bottom) *L*_3_/*L*_2_ ratio in Co *L*_2,3_ edge, which were reconstructed by NLLS fitting process for the obtained EELS spectrum image dataset. f) Several O K and Co *L*_2,3_ EELS profiles were extracted from five different regions randomly chosen for the HCO film.

To examine the morphological structure of the grown thin films and verify whether Co atoms were intentionally doped in the parent HfO_2_ films, EDX mapping in high‐angle annular dark field scanning transmission electron microscopy (HAADF STEM) imaging mode was performed. **Figure** **3**a shows elemental maps of Hf, Co, O, and Si in the HCO‐30 film grown on a silicon substrate. A low‐magnification HAADF STEM image of the HCO‐30 film confirms the film to be ultrathin with a thickness of ≈5 nm and a smooth surface. Note that heavy elements such as Hf and Co atoms are visible while light elements, such as Si and O atoms are barely imaged because the image contrast is scaled to atomic number squared in this imaging mode.^[^ ^36^ ^]^ EDX elemental maps of Hf (pink) and Co (yellow) atoms show that Co atoms are evenly distributed over the entire thin film. This uniform distribution of Co atoms was also demonstrated in the HCO‐10 film (Figure S4, Supporting Information). The EDX map of O (green) atoms represents the presence of an amorphous SiO_2_ bottom layer on the Si substrate, which is generally formed due to the interface reaction during HfO_2_ film growth.^[^ ^37^ ^]^ The SiO_2_ interlayer formation induces the subsequently upper‐grown HfO_2_ film to be intrinsically oxygen deficient,^[^ ^37^ ^]^ which is supported by the XPS analysis of the O 1*s* core level in the pristine HfO_2_. The origin of the SiO*_x_* layer is mainly during the solution‐phase deposition and annealing step. Oxygen comes out from HCO and reacts to form low permittivity amorphous dielectric.^[^ ^37^, ^38^ ^]^ Comparison of the EDX spectra of Co peaks normalized to the Hf peak intensity shows the increased Co dopants from HCO‐10 to HCO‐30 samples (Figure 3b). In the GIXRD results, it was hard to determine the crystal quality and phases of the three grown films owing to their thin thickness at 5 nm. Thus, we employed atomic resolution HAADF STEM imaging to observe their real lattice structures. As a result, we see that all the samples showed well‐developed crystal structures but different phase configurations. The HCO‐10 and 30 samples (10 and 30 indicate the mass of cobalt nitrate hexahydrate to synthesize the corresponding HCO solution was 11 and 30 mg, respectively) were a mixture of nonpolar monoclinic (*P*2_1_/*c*) and polar orthorhombic (*Pca*2_1_) phases (see the representative STEM images in Figure 3c,d. The orthorhombic HfO_2_ phase was known to be metastable at room temperature but stabilized by introducing oxygen vacancies therein.^[^ ^39^ ^]^ Considering the vacancy‐induced phase transition, previous theoretical calculations revealed that a threshold content of oxygen vacancies triggering the monoclinic to orthorhombic phase transition could reach more than ≈20%. This result implies that the HfO_2_ lattice has robust stability for a wide range of defect densities without structural collapse.^[^ ^37^ ^]^ The oxygen vacancies in the HCO films aid in glucose sensing and also promote phase transformation from nonpolar monoclinic to polar orthorhombic. This suggests another potential application of these films as nonperovskite ferroelectrics on silicon at smaller scales. However, more research is needed to understand their polarization behavior. Further, the notable tolerance for bearing a high density of defects can be a key parameter to tailor the electronic structure of the HfO_2_ thin film for defect engineering. These results agree well with previous results for HfO_2_ thin films doped with other elements such as Zr and La.^[^ ^37^, ^40^ ^]^ In contrast, the nonpolar monoclinic phase, which is thermodynamically stable at room temperature, was only observed in the pristine HfO_2_ sample (Figure S5, Supporting Information). Since the orthorhombic phase of HfO_2_ exhibited a distorted crystal structure with a permanent dipole moment, this structural characteristic could form more active sites on the surface and thus facilitate the adsorption and activation of reactant molecules, contributing to the catalytic reaction.

Co dopants are popularly used to enhance the catalytic activity of the host‐HfO_2_ material because they can exist in multivalence states, boosting the electron transfer process and oxygen vacancy formation. To confirm the increase of oxygen vacancy content associated with their multivalence state, we examined changes in the A/B ratio of O *K* edge and the *L*_3_/*L*_2_ ratio of Co *L*_2,3_ edge obtained using electron energy loss spectroscopy spectrum imaging (EELS SI) (Figure 3e,f). In O *K* edge of HfO_2_, the doublet‐peak shape is notably observed, which is denoted as A (at ≈534 eV) and B (at ≈538 eV) and attributed to the O 2*p* orbitals hybridized with Hf $d_{e_{g}}$ and $d_{t_{2g}}$ orbitals, respectively.^[^ ^41^ ^]^ A decrease in the relative intensity of A compared to that of B is correlated with the increase in defect densities and local structural disorder, thus generally using the A/B spectral signature as an indicator for monitoring the change in vacancy content.^[^ ^37^, ^42^ ^]^ The A/B ratio map and the representative O *K* edge profiles extracted from EELS SI dataset for the HCO‐30 sample are displayed in Figure 3e (top) and 3f (left). Compared to the averaged A/B ratio (≈0.78) of the pristine HfO_2_ (see full dataset given in Figure S6, Supporting Information), that of the HCO‐30 sample was measured to be reduced to ≈0.72, supporting the increase of oxygen vacancy concentration by Co doping. The A/B ratio map for O *K* edge indicates that oxygen vacancies are randomly distributed over the film, which is correlated to the uniform distribution of Co dopants confirmed by EDX mapping. For Co *L*_2,3_ edge, the valence state of Co can be interpreted by measuring the *L*_3_/*L*_2_ ratio because the intensity ratio of *L*_3_ over *L*_2_ peaks and the position of *L*_2,3_ edges in EEL spectra of 3*d* elements sensitively change with the oxidation state and local bonding variation.^[^ ^43^ ^]^ In the case of Co, the value of the *L*_3_/*L*_2_ ratio increases as the valence state of Co decreases, and the onset energy of the edges increases, as tested with the standard samples (Figure S7, Supporting Information). In this, measuring the *L*_3_/*L*_2_ ratio is preferred over measuring the onset energy change because the onset energy measurement requires careful energy calibration and high energy resolution to discriminate the slight shift in energy difference. Hence, we obtained an *L*_3_/*L*_2_ ratio map calculated from the Co *L*_2,3_ ELNES SI dataset as shown in Figure 3e (bottom), and the representative Co *L*_2,3_ edges randomly extracted from the SI dataset are represented in Figure 3f (right) to verify the multivalency of Co dopants in the HCO film. The result indicates that the averaged *L*_3_/*L*_2_ ratio is estimated at ≈2.5. This value corresponds to the typical value indicating the mixed valence state of Co^2+3+^ (Figure S7, Supporting Information), demonstrating that Co dopants are randomly distributed over the HCO film in the multivalence state. It should be noted that the actual doping content of Co was revealed to be roughly half of the designed doping composition (Figure S5, Supporting Information). Therefore, due to the small content, EELS SI could not reliably detect Co dopants of less than 10% distributed in HCO‐10 film.

The structural and chemical characteristics of the HCO thin film suggest how much the CSD‐HCO‐based electrode can help create a stable catalyst system with high activity for the electrochemical sensing of glucose. From a chemical point of view, adding Co dopants with multiple oxidation states for a wide range of concentrations should help create electrochemically active sites on the surface of the HfO_2_ lattice, which can facilitate redox reactions involving electron transfer processes.^[^ ^44^ ^]^ In association with the Co doping, the oxygen vacancy content was further increased in the host HfO_2_ structure, expecting to promote oxidation reactions. Meanwhile, from the structural perspective of the host material, the HfO_2_ with various polymorphs and distorted metastable phases that are close in energy^[^ ^45^ ^]^ can be maintained stable for a wide range of defect densities and their frequent in‐and‐out inside the lattice without structural collapse.^[^ ^46^ ^]^ Of polymorphic phases, the polar orthorhombic phase stabilized by the increased oxygen vacancy content is expected to favor further enhancing the surface catalytic activities. Therefore, the exceptional structural stability of HfO_2_ should be considered a pivotal attribute required for fabricating a high‐performance, durable catalyst system for glucose sensing.

To elucidate the effect of Co doping on the enhanced electrocatalytic activity in HCO, we investigate the role of oxygen vacancy using first‐principles density functional theory (DFT) calculations. We considered the oxygen vacancies in the monoclinic HfO_2_ (*m‐*HfO_2_), orthorhombic HfO_2_ (*o‐*HfO_2_), Co‐doped *m‐*HfO_2_ (*m*‐HCO), and Co‐doped *o‐*HfO_2_ (*o*‐HCO), and calculated the oxygen vacancy formation energy for all possible oxygen vacancy sites. As illustrated in Figure S8a (Supporting Information), there are two potential oxygen vacancy sites, O_I_ and O_II_, in *m*‐HfO₂ and *o*‐HfO₂, depending on the relative coordinates with respect to the surrounding Hf cations. Here, O_I_ and O_II_ indicate oxygen atoms with three and four nearest‐neighboring Hf cations, respectively. In the case of HCO, there are four possible oxygen vacancy sites, O_1_, O_2_, O_3,_ and O_4_, as shown in Figure S8b (Supporting Information). Here, O_1_ atoms have three nearest‐neighboring Hf cations and one Co cations. O_2_ atoms have two nearest‐neighboring Hf and one Co cation. O_3_ and O_4_ atoms have three and four nearest‐neighboring Hf cations, respectively.

**Figure 4:**
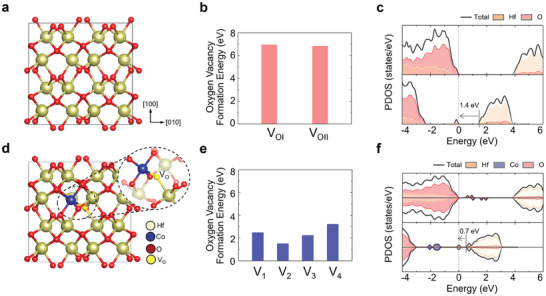
Theoretical calculations of oxygen vacancy formation in HCO. a,d) Crystal structures of *m*‐HfO_2_ and *m*‐HCO. b,e) Oxygen vacancy formation energies in *m*‐HfO_2_ and *m*‐HCO with possible oxygen vacancy sites. c,f) Density of states for *m*‐HfO_2_ and *m*‐HCO without oxygen vacancies (top panel) and with oxygen vacancies (bottom panel). The Fermi level is set as zero.

The oxygen vacancy formation energy was defined as

(2) $$E_{\mathrm{vac}}=E\left({\mathrm{HfO}}_{2}+V_{O}\right)-E\left(\mathrm{Hf}O_{2}\right)+\frac{1}{2}E\left(O_{2}\right)$$

where *E*(HfO_2_ + *V*_O_) and *E*(HfO_2_) are the total energy of the defective structures including oxygen vacancies, and that of the pristine structures without oxygen vacancies, respectively. *E*(O_2_) is the energy of an O_2_ molecule. The oxygen vacancy formation energies for O_I_ and O_II_ in *m‐*HfO_2_ are estimated to be ≈7 eV, which is relatively high compared to other oxide materials.^[^ ^47^ ^]^ In contrast, when the Co dopant is introduced into the *m‐*HfO_2_ structure, the oxygen vacancy formation energies are substantially reduced regardless of oxygen sites. In particular, the vacancy formation energy for the O_2_ site, which has two nearest‐neighboring Hf and one Co cation, is dramatically reduced from ≈7 to 1.5 eV (**Figure** **4**e), indicating that substitutional Co doping significantly facilitates the formation of oxygen vacancies. This formation energy is much lower than that of pristine Co_3_O_4_ (≈3.2 eV),^[^ ^47d^ ^]^ which has been widely used as a sensor material. As shown in Figure S9 (Supporting Information), we also observed a great reduction in the oxygen vacancy formation energies in the *o‐*HCO structure (from ≈7 to ≈3 eV). To further investigate the changes in the electronic structure caused by oxygen vacancies, we calculated the density of states for the *m‐*HfO_2_ and the *m‐*HCO, considering oxygen vacancies at the most energetically probable sites. As shown in Figure 4c top panel, the bandgap of pristine *m‐*HfO_2_ was calculated to be ≈4 eV. When the oxygen vacancy is introduced *m‐*HfO_2_ structure (Figure 4c bottom panel), a defect state is formed 1.4 eV away from the conduction band minimum. In contrast, when oxygen vacancy is introduced in *m‐*HCO (Figure 4e bottom panel), a shallow donor level is formed ≈0.7 eV away from the conduction band minimum. This shallow donor state facilitates electron donation to the conduction band, leading to enhanced electrocatalytic activity. Consequently, we confirmed that oxidation reactions occur more readily in *m*‐HCO than in pure *m*‐HfO₂. Our DFT calculations demonstrate that oxygen vacancies are readily generated in HCO, and the resulting shallow donor level significantly enhances electrocatalytic activity by more efficiently promoting charge transfer to the conduction band.

The electrocatalytic performance of pristine HfO_2_ and HCO in the absence and presence of glucose were investigated in the 0.1 m NaOH electrolyte at the scan rate of 10 mV s^−1^ to confirm the active site for oxidation of glucose (Figure 1d). In the case of HCO‐10 in 10 mm glucose with alkaline solution (solid line, purple in color), two substantial peaks were observed in the forward scan (at +0.25 V) and the reverse scan (at +0.1 V). The former peak indicates oxidation of glucose because no peak is observed in 0 mm glucose with an alkaline solution (blank solution, dotted line, light purple in color presenting pivotal role of Co in HCO. According to the proposed mechanism of glucose electrooxidation by the nonenzymatic material, conversion of glucose is done by following steps: 1) adsorption of glucose to the electrode surface and 2) glucose is oxidized to the adsorbed gluconolactone intermediate, catalyzed by OH ions which strongly adsorb to the surface of electrode and mediate the conversion of glucose.^[^ ^48^ ^]^ It has been reported that the transition metal ions such as Co^2+^ in Co_3_O_4_ catalyze glucose electrooxidation by the following equations^[^ ^49^ ^]^

(3) $$Co_{3}O_{4}+OH^{-}+H_{2}O↔3\mathrm{CoOOH}+e^{-}$$

(4) $$\mathrm{CoOOH}+OH^{-}\to \mathrm{Co}O_{2}+H_{2}O+e^{-}$$

(5) $$\begin{array}{ccc} 2\mathrm{Co}O_{2} & + & C_{6}H_{12}O_{6}\left(\mathrm{glucose}\right)\to 2\mathrm{CoOOH}\\ & + & C_{6}H_{10}O_{6}\left(\mathrm{gluconolactone}\right) \end{array}$$

**Figure 5:**
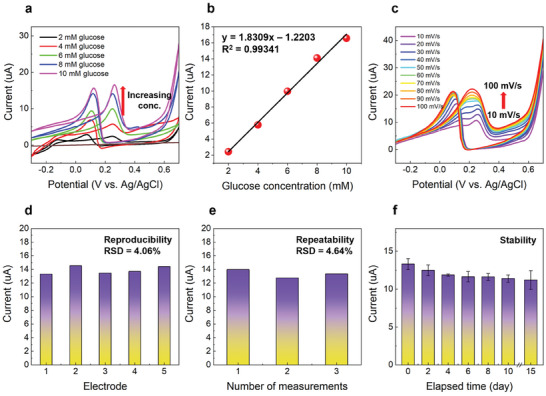
Performance of HCO electrodes in nonenzymatic glucose sensing in 0.1 m NaOH. a) CV response at different glucose concentrations from 2 to 10 mm. b) Corresponding calibration plot of peak current versus glucose concentration. c) CV response of HCO‐10 of 10 mm in 0.1m NaOH at different scan rates from 10 to 100 mV s^−1^. d) Reproducibility test for five HCO‐10 electrodes. e) Repeatability test of HCO‐10 for three consecutive measurements. f) Stability test of HCO‐10 over a period of 10 days.

It was reported that not only Co^2+^ but also Co^3+^ plays a key role in glucose electrooxidation.^[^ ^44^ ^]^ Co^3+^ is responsible for the formation of surface double‐layer capacitance (DLC) and the surface DLC leads to the accumulation of hydroxyl ions, promoting the whole electrocatalytic reaction.^[^ ^50^ ^]^ Therefore, the glucose electrooxidation phenomenon of HCO is attributed to the mixed valence state of Co observed in the Co *L*_2,3_ EELS profiles (Figure 3e) and hydroxyl ions. In this regard, under neutral conditions such as phosphate‐buffered saline (PBS) at pH 7.4, which lacks hydroxyl groups, HCO exhibits limitations in performing effectively as a glucose sensor, as it would in alkaline environments (Figure S10, Supporting Information). In order to evaluate the linear response of HCO with respect to various glucose concentrations, CV scans were done at different glucose concentrations from 2 to 10 mm, as shown in **Figure** **5**a. As the glucose concentrations increased from 2 to 10 mm, corresponding peaks related to the adsorption of glucose to the electrode surface and oxidation of glucose into gluconolactone increased monotonically. To evaluate whether HCO material meets the criteria of continuous monitoring for glucose in alkaline media, the peak current versus glucose concentration is plotted as shown in Figure 5b. It has a linear dynamic range within 2–10 mm glucose levels, with the coefficient of determination *R* ^2^ = 0.99 341. Since diabetes mellitus refers to higher or lower blood glucose concentrations than the normal range of 4.4–6.6 mm,^[^ ^51^ ^]^ the linear response of HCO electrodes between 2 and 10 mm glucose levels could satisfy the requirements of continuous blood glucose monitoring performances. From the fitting data, the limit of detection (LoD) can be calculated by the following equation^[^ ^52^ ^]^

(6) LoD=3.3σ/S

where *σ* is the standard deviation of the background current, while *S* is the slope of the linear calibration curve measured by the glucose sensor. The detection limit was estimated to be 0.46 mm glucose in 0.1 m NaOH solution. The sensitivity of the sensor was calculated as the ratio of the slope to the electrode area,^[^ ^53^ ^]^ which is to be 1.831 µA mm ^−1^ cm^−2^. Such low values of LoD and sensitivity are thought that the HCO electrode has a flat surface. To figure out the limiting step of the electrochemical reaction in the conversion of glucose, the effects of scan rate for HCO‐10 were tested at different scan rates from 10 to 100 mV s^−1^ in the presence of 10 mm glucose in 0.1 m NaOH as shown in Figure 5c. The fitting data was plotted in Figure S11 (Supporting Information) and the peak current was proportional to the square root of the scan rate (*v* ^1/2^), suggesting that the rate‐determining step is the diffusion of the glucose to the electrode surface, governed by the concentration of glucose. The selectivity of a glucose sensor is crucial to ensure its responsiveness specifically to glucose, excluding interference from species like ascorbic acid (AA), dopamine (DA), and uric acid (UA), etc. These interference species exist in physiological fluids, posing challenges for the direct electrochemical oxidation of glucose on sensing materials, particularly nonenzymatic sensors.^[^ ^54^ ^]^ To evaluate the selectivity of CSD‐HCO as the nonenzymatic glucose sensing material, the normal physiological levels (following concentrations are based on volume after injection) of glucose (10 mm), AA (0.1 mm), DA (0.1 mm), UA (0.1 mm), NaCl (0.1 mm), lactose (0.1 mm), and sucrose (0.1 mm) were orderly injected into 0.1 m NaOH at +0.25 V by chronoamperometric measurements. As depicted in Figure 1e, HCO exhibited exceptional selectivity against interference species, with a substantial increase in current observed only after the injection of glucose, not with major electro‐active species. This suggests that HCO is suitable for accurately determining glucose concentration in the presence of other interference species. Also, current increased only after injection of glucose and the current value doubled within 2 s compared to initial value of current, as shown inset of Figure 1e. Such rapid response with respect to change in glucose plays a crucial role for continuous glucose monitoring system. To assess reproducibility, peak current values corresponding to glucose oxidation in the CV response of five different electrodes were recorded under optimized conditions (Figure S12, Supporting Information). Figure 5d shows a relative standard deviation (RSD) of 4.06%, indicating good reproducibility. The repeatability was assessed similar to reproducibility, involving three repeated measurements by a single electrode within a day. Figure 5e; and Figure S13 (Supporting Information) displays a RSD of 4.64%, demonstrating good repeatability of the proposed sensor. Stability was evaluated by periodically measuring the response current to 10 mm glucose every 2 days, as depicted in Figure 5f; and Figure S14 (Supporting Information). After storing the electrode in the air for 15 days, the response current retained ≈84.1% of its initial value, indicating good storage stability.

## 3 Conclusion

In summary, we demonstrated that CSD‐processed HCO thin films exhibit nonenzymatic glucose detection performance. As some Hf atoms in HfO_2_ are substituted by Co atoms with multivalence states, Co acts as an active site that oxidizes glucose present in the solution. Additionally, the substitution of some Hf atoms with Co reduces the energy required for the formation of oxygen vacancies in the thin film, leading to an increase in oxygen vacancies. This increase contributes to enhanced oxidation reactions of glucose at the surface in HCO compared to HfO_2_. Aqueous‐based CSD facilitates the direct formation of HCO on Si/SiO*_x_*, making it suitable for fabricating integrated glucose detection sensor devices. Consequently, the glucose oxidation activity of Co atoms with multivalence states and the synergistic effect of them for nonenzymatic glucose detection have been experimentally demonstrated. HCO films demonstrate considerable selectivity, repeatability, and reproducible sensing characteristics. The presence of multivalent Co in HCO is crucial for achieving high‐performance glucose sensing.

## 4 Experimental Section

### Materials

Hafnium (IV) chloride (HfCl_4_), cobalt (II) nitrate hexahydrate (Co(NO_3_)_2_), 2‐Methoxyethanol (2‐ME), D‐(+)‐glucose, and phosphate buffered saline (PBS) were purchased from Sigma‐Aldrich and 0.1 m sodium hydroxide (NaOH) standard solution from Daejung Chemicals & Metals Co., Ltd. All reagents were used without further purification.

### Synthesis of CSD Precursor and Deposition of Thin Films

To synthesize HCO‐10 precursor solution, 0.2 g of HfCl_4_, and 10 mg of Co(NO_3_)_2_ were dissolved in 5 mL of deionized (DI) water and 3 mL of 2‐ME, respectively. Then, both solutions were mixed homogeneously and stirred at room temperature for 12 h. Later, the HCO‐10 solution was spin‐coated on a heavily doped p‐type Si substrate (cleaned in the order of buffered oxide etchant (1:40), DI water, acetone, isopropyl alcohol, and DI water) with 10 s ramp and 3000 rpm for 30 s. The as‐spun films were thermally annealed at 350 °C for 2 h in the open air to render HCO and oxygen vacancies form and then cooled down to room temperature. Subsequently, they were treated with rapid thermal annealing (RTA) at 700 °C with a heating rate of 10 °C s^−1^ for 1 min to stabilize the metastable state of HCO film. In the case of HCO‐30 precursor solution, 30 mg of Co(NO_3_)_2_ was dissolved in 5 mL of DI water.

### Characterization of CSD Precursor and HCO Thin Films

Thermogravimetry/differential thermal analyzer (TG/DTA, Seiko Exstar 6000) was used to investigate the thermal behavior of HCO precursor at a heating rate of 10 °C min^−1^. The structural characterization of thin films was analyzed by grazing incidence X‐ray diffraction (GIXRD, D8 ADVANCE) using Cu Kα radiation (wavelength: 1.5406 Å) with a 0.3° angle of incidence. Cross‐sectional samples for the STEM experiment were prepared using a focused ion beam (FIB, FEI Helios NanoLab 450 S) milling technique. Subsequently, atomic‐scale and nanoscale structures of the HfO_2_ and HCO samples were observed by aberration‐corrected STEM (GrandARM300CF, JEOL) operating at 300 kV with a semiconvergence angle of ≈32 mrad. Atomic‐resolution images were obtained using high‐angle annular dark field (HAADF) imaging mode with a detector angle range of 79–180 mrad. Energy‐dispersive X‐ray spectroscopy (EDX, JEOL JED100) was employed for nanoscale elemental distribution mapping of the samples. A silicon drift detector (SDD) with a large effective X‐ray sensing area of 100 mm^2^ was used within the STEM for EDX mapping. Electron energy loss spectroscopy (EELS, Gatan GIF Quantum ER 965) spectrum imaging (SI) was used to analyze the energy‐loss near‐edge structure (ELNES) at the K edge of O and the L edge of Co in the samples. EELS SI data were acquired for the samples using a 5 mm entrance aperture. The data have an energy dispersion of 0.25 eV ch^−1^ and an energy loss range of 470–982 eV. To quantify the O A/B ratio and the Co *L*_3_/*L*_2_ ratio, a nonlinear least‐squares (NLLS) curve fitting method was applied to each peak of the O K edge and Co L edge spectra, respectively. NLLS curve fitting was performed using Gatan Digital Micrograph software. Besides, X‐ray photoelectron spectroscopy (XPS, ESCALAB250) was used to estimate the chemical bonding states and stoichiometry of pristine HfO_2_ and HCO films.

### Density Functional Theory Calculation

All calculations to obtain electronic structures and the formation energy of an oxygen vacancy were carried out using the projector augmented wave (PAW) method with plane‐wave‐based Vienna ab initio simulation package (VASP).^[^ ^55^, ^56^ ^]^ The generalized‐gradient approximation (GGA) with the Perdew–Burke–Ernzerhof (PBE) functional for the exchange‐correlation functional was used.^[^ ^57^ ^]^ An effective Hubbard parameter *U*_eff_ =  U − J with 3 eV for the localized Co d electrons was applied. A 2 × 2 × 2 supercell of HfO_2_ was utilized to consider the Co dopant and the formation of oxygen vacancy. A Γ‐centered 4 × 4 × 4 *k*‐point mesh was used for the oxygen vacancy formation energy calculation and plane waves with a kinetic energy cutoff of up to 500 eV were included. The energy convergence tolerance for all atoms was set to 10^−6^ eV atom^−1^, and the system was fully relaxed until the final force reached 0.01 eV Å^−1^.

### Fabrication of Nonenzymatic Glucose Sensing Electrode

For the preparation of the working electrode (WE), a 1 cm^2^ area of the HCO film was coated with 20 nm of Ti followed by 100 nm of Au using e‐beam evaporation. The remaining 1 cm^2^ of the film was covered with a polyethylene terephthalate (PET) film mask to keep it exposed for contact with the glucose solution electrolyte. To ensure the electrochemical reaction is not limited by the WE's surface area, the contact area of the WE with the glucose solution should be comparable to or smaller than the 1 cm^2^ platinum counter electrode (CE).

### Electrochemical Measurement

All electrochemical measurements were performed at room temperature in either 0.1 m NaOH electrolyte solution or 0.01 m phosphate buffered saline (PBS). The results were collected using an Ivium‐n‐Stat electrochemical analyzer (Ivium Technologies). A standard three‐electrode system was used, comprising an HCO working electrode (1 cm^2^), a platinum plate counter electrode (1 cm^2^), and an Ag/AgCl reference electrode with a 1 m KCl internal solution (ALS Co., Ltd). Note that the first CV cycle is not taken into consideration because it is used for stabilizing the current. The average calculations for repeatability, reproducibility, and stability tests are based on data from the second to the fifth cycle.

### Statistical Analysis

Data obtained from the experiments were subjected to statistical analysis, ensuring a reliable interpretation of the results. All data underwent preprocessing procedure, including normalization, transformation, and discarding outliers in advance of statistical analysis. Outliers were identified and evaluated using appropriate statistical methods, considering other data except for outliers. Data are presented as mean ± standard deviation (SD) with sample sizes (*n*) were indicated for each statistical analysis. Data used for statistical analysis were randomly chosen by double‐blind test to ensure the validity of assumptions at the 5% level (*P* value = 0.05). Statistical analysis was performed using SAS 9.4 software (SAS institute, NC).

## Conflict of Interest

The authors declare no conflict of interest.

## Author Contributions

J.O., A.S.H.W., E.‐B.P., and J.H. contributed equally to this work. J.O., A.S.H.W., M. T. K., and P.P. conceptualized the study. J.O. and A.S.H.W. conducted the TG/DTA, XRD, and XPS analyses, carried out the electrochemical measurements, and analyzed the glucose sensing data. M.T.K. conducted the fabrication of glucose detection sensors and deposition the electrodes for the electrochemical measurements. P.P. and S.K.K. supervised the XPS and glucose sensing studies. E.B.P. performed STEM/EDX and EELS analyses. Y.M.K. supervised the STEM/TEM studies. S.J.K. conducted FIB TEM sampling, and H.Y.J. supervised TEM sampling. J.H. performed DFT calculations, and J.L. supervised the DFT studies. All authors contributed to writing, reviewing, and approving the final version of the manuscript.

## Supporting information



Supporting Information

## Data Availability

Research data are not shared.
