# Supplementary material for: Enhancing Nonenzymatic Glucose Detection Through Cobalt‐Substituted Hafnia
Source: Adv Sci (Weinh). 2025 Feb 24;12(15):2408687. doi: 10.1002/advs.202408687 (PMC12005825; doi:10.1002/advs.202408687)
Supplement: Supplementary file 1 — Supporting Information [file ADVS-12-2408687-s001.docx]

**Supporting Information**

for

**Enhancing Non-Enzymatic Glucose Detection through Cobalt-Substituted Hafnia**

*Jeonghyeon Oh^1,#^, Avis Sin Hui Wee^1,#^, Eun-Byeol Park^2,#^, Jaejin Hwang^3,#^, Seon Je Kim^2^, Hu Young Jeong^4^, Myat Thet Khine^1^, Pavan Pujar^5,*^, Jaekwang Lee^3,*^, Young-Min Kim^2,*^, and Sunkook Kim^1,*^*

^1^Multifunctional Nano Bio Electronics Lab, School of Advanced Materials Science and Engineering, Sungkyunkwan University, Gyeonggi-do, Suwon 16419, Republic of Korea

^2^Department of Energy Science, Sungkyunkwan University (SKKU), Gyeonggi-do, Suwon 16419, Republic of Korea

^3^Department of Physics, Pusan National University, Busan 46241, Republic of Korea

^4^Graduate School of Semiconductor Materials and Devices Engineering, Ulsan National Institute of Science and Technology (UNIST), Ulsan 44919, Republic of Korea

^5^Department of Ceramic Engineering, Indian Institute of Technology (IIT-BHU), Varanasi, Uttar Pradesh 221005, India

^#^These authors contributed equally to this work.

*Corresponding authors: Pavan Pujar ([pavan.cer@iitbhu.ac.in](mailto:pavan.cer@iitbhu.ac.in)), Jaekwang Lee ([jaekwang1@pusan.ac.kr](mailto:jaekwang1@pusan.ac.kr)), Young-Min Kim ([youngmk@skku.edu](mailto:youngmk@skku.edu)), Sunkook Kim ([seonkuk@skku.edu](mailto:seonkuk@skku.edu))


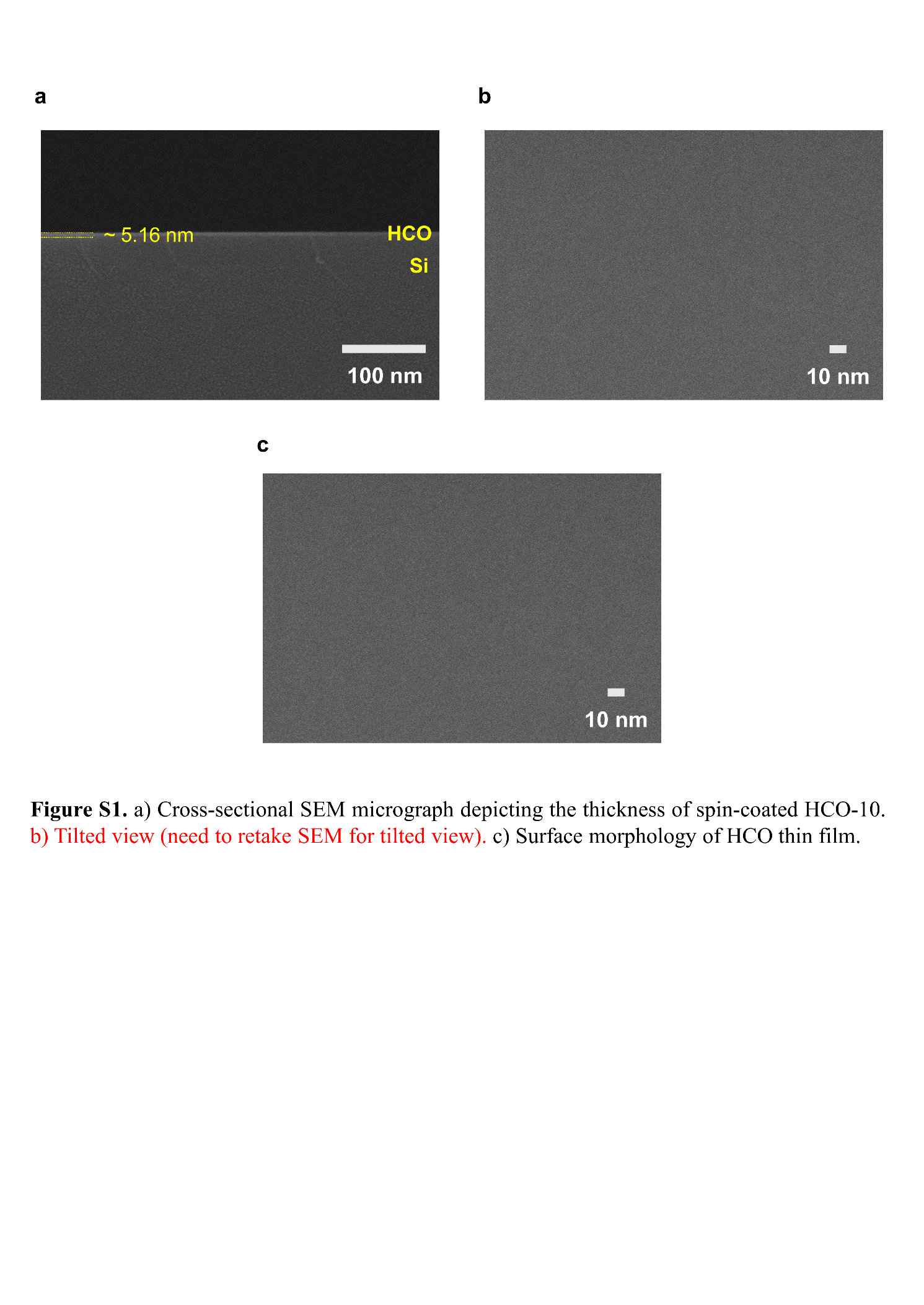


**Figure S1.** Cross-sectional SEM micrograph depicting the thickness of spin-coated HCO-10.


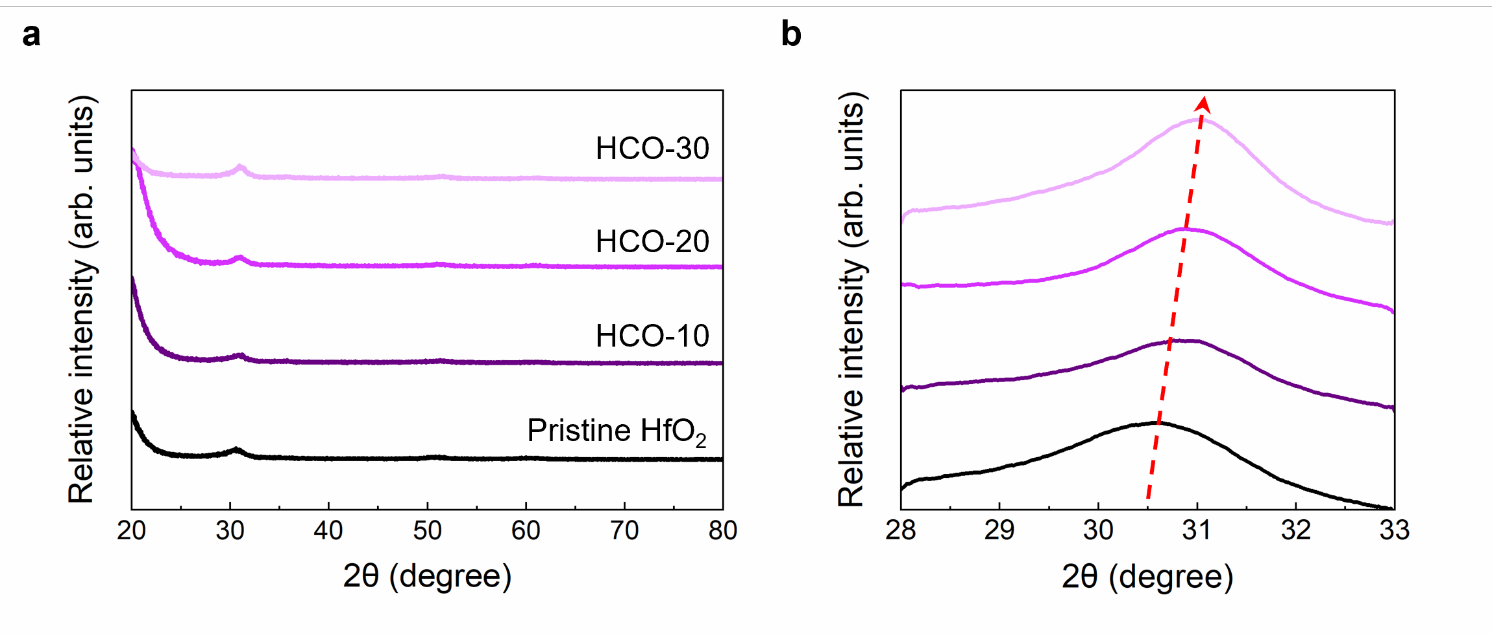


**Figure S2.** Structural characterization of CSD-HCO films grown on silicon. a) GIXRD patterns and b) enlarged view of CSD-HCO films at different Co doping concentrations.

**Table S1.** FWHM, d-spacing and crystallite size of HCO thin films at different Co doping concentrations.

| Sample | 2θ [°] | FWHM | d-spacing [Å] | Crystallite size [nm] |
| --- | --- | --- | --- | --- |
| Pristine HfO_2_ | 30.6 | 2.5819 | 1.5132 | 3.2 |
| HCO-10 | 30.76 | 2.4156 | 1.5061 | 3.4 |
| HCO-20 | 30.88 | 1.9060 | 1.5009 | 4.3 |
| HCO-30 | 31 | 1.6726 | 1.4956 | 4.9 |


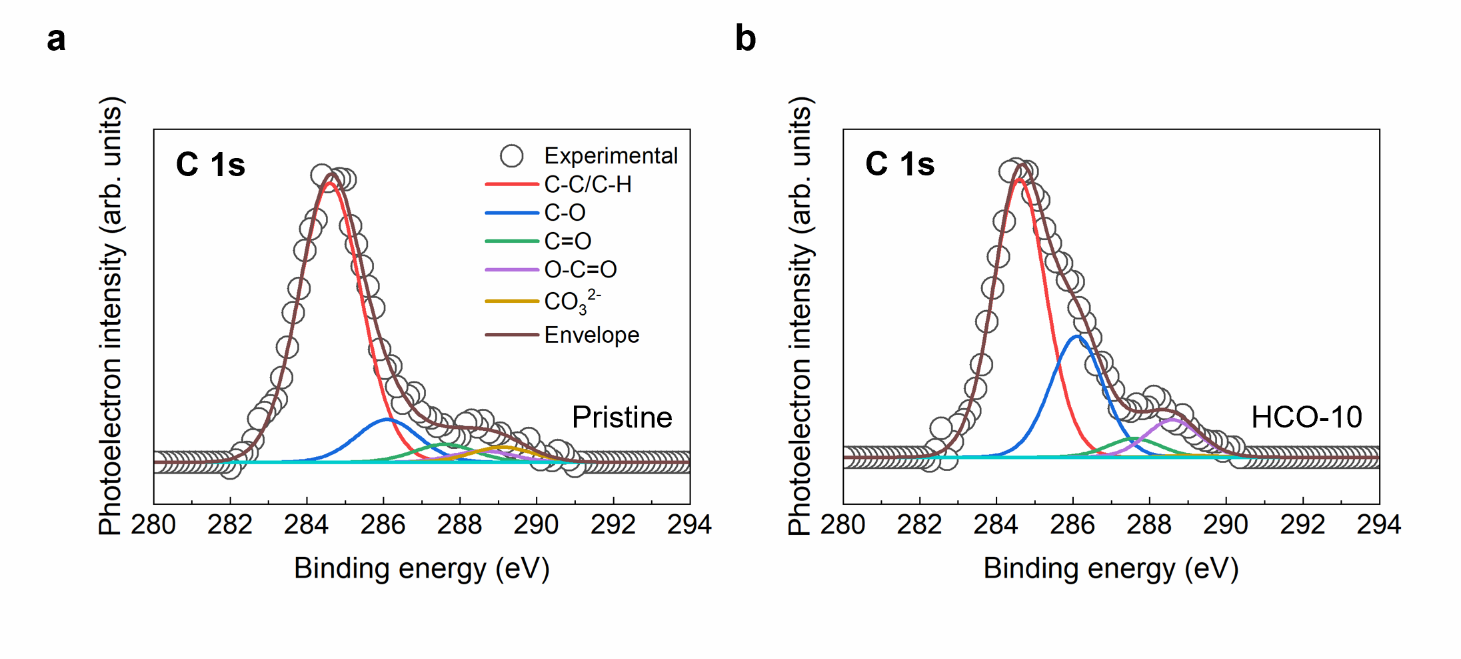


**Figure S3.** High-resolution deconvoluted C 1s spectra for a) pristine HfO_2_ and b) HCO-10.

**Table S2a.** Peak fitting parameters of C 1s for pristine HfO_2_.

| Peak signature | Position constraint | Position [eV] | FWHM | Area | Atomic % |
| --- | --- | --- | --- | --- | --- |
| C-C/C-H  (Peak I) | - | 284.60 | 1.92 | 13982.08 | 75.99 |
| C-O  (Peak II) | Peak I + 1.5 eV | 286.10 | 1.92 | 2155.53 | 11.72 |
| C=O  (Peak III) | Peak I + 3.0 eV | 287.60 | 1.92 | 909.05 | 4.94 |
| O-C=O  (Peak IV) | Peak I + 4.0 eV | 288.60 | 1.92 | 577.50 | 3.14 |
| CO^3^_2-_  (Peak IV) | Peak I + 4.5 eV | 289.10 | 1.92 | 775.49 | 4.21 |

**Table S2b.** Peak fitting parameters of C 1s for HCO-10.

| Peak signature | Position constraint | Position [eV] | FWHM | Area | Atomic % |
| --- | --- | --- | --- | --- | --- |
| C-C/C-H  (Peak I) | - | 284.60 | 1.63 | 10481.37 | 60.82 |
| C-O  (Peak II) | Peak I + 1.5 eV | 286.10 | 1.63 | 4563.19 | 26.48 |
| C=O  (Peak III) | Peak I + 3.0 eV | 287.60 | 1.63 | 709.53 | 4.12 |
| O-C=O  (Peak IV) | Peak I + 4.0 eV | 288.60 | 1.63 | 1416.04 | 8.22 |
| CO^3^_2-_  (Peak IV) | Peak I + 4.5 eV | 289.10 | 1.63 | 62.32 | 0.36 |

**Table S2c.** Peak fitting parameters of Hf 4f and O 1s for pristine HfO_2_.

| Peak signature | Position [eV] | FWHM | Area | Atomic % |
| --- | --- | --- | --- | --- |
| Hf 4f_7/2_ | 15.53 | 1.13 | 110204.47 | 40.76 |
| Hf 4f_5/2_ | 17.21 | 1.13 | 87015.35 | 32.18 |
| Hf-O-Si | 16.44 | 1.13 | 42702.61 | 15.79 |
| Hf-O-Si | 18.13 | 1.13 | 30469.44 | 11.27 |
| M-O-M | 529.29 | 1.59 | 195555.62 | 79.88 |
| O-Vacancy | 531.13 | 1.59 | 49255.44 | 20.12 |

**Table S2d.** Peak fitting parameters of Hf 4f, O 1s and Co 2p for HCO-10.

| Peak signature | Position [eV] | FWHM | Area | Atomic % |
| --- | --- | --- | --- | --- |
| Hf 4f_7/2_ | 16.52 | 1.12 | 143116.95 | 42.20 |
| Hf 4f_5/2_ | 18.20 | 1.12 | 113296.44 | 33.41 |
| Hf-O-Si | 17.53 | 1.12 | 48185.92 | 14.21 |
| Hf-O-Si | 19.23 | 1.12 | 34545.17 | 10.19 |
| M-O-M | 530.09 | 1.51 | 261655.15 | 77.00 |
| O-Vacancy | 532.06 | 1.51 | 78157.95 | 23.00 |
| Co 2p_3/2_ | 781.32 | 3.99 | 13664.51 | 42.41 |
| Co 2p_1/2_ | 796.31 | 3.99 | 6540.05 | 20.30 |
| Co 2p_3/2_ satellite | 796.81 | 3.99 | 7542.04 | 23.41 |
| Co 2p_1/2_ satellite | 802.39 | 3.99 | 4474.97 | 13.89 |


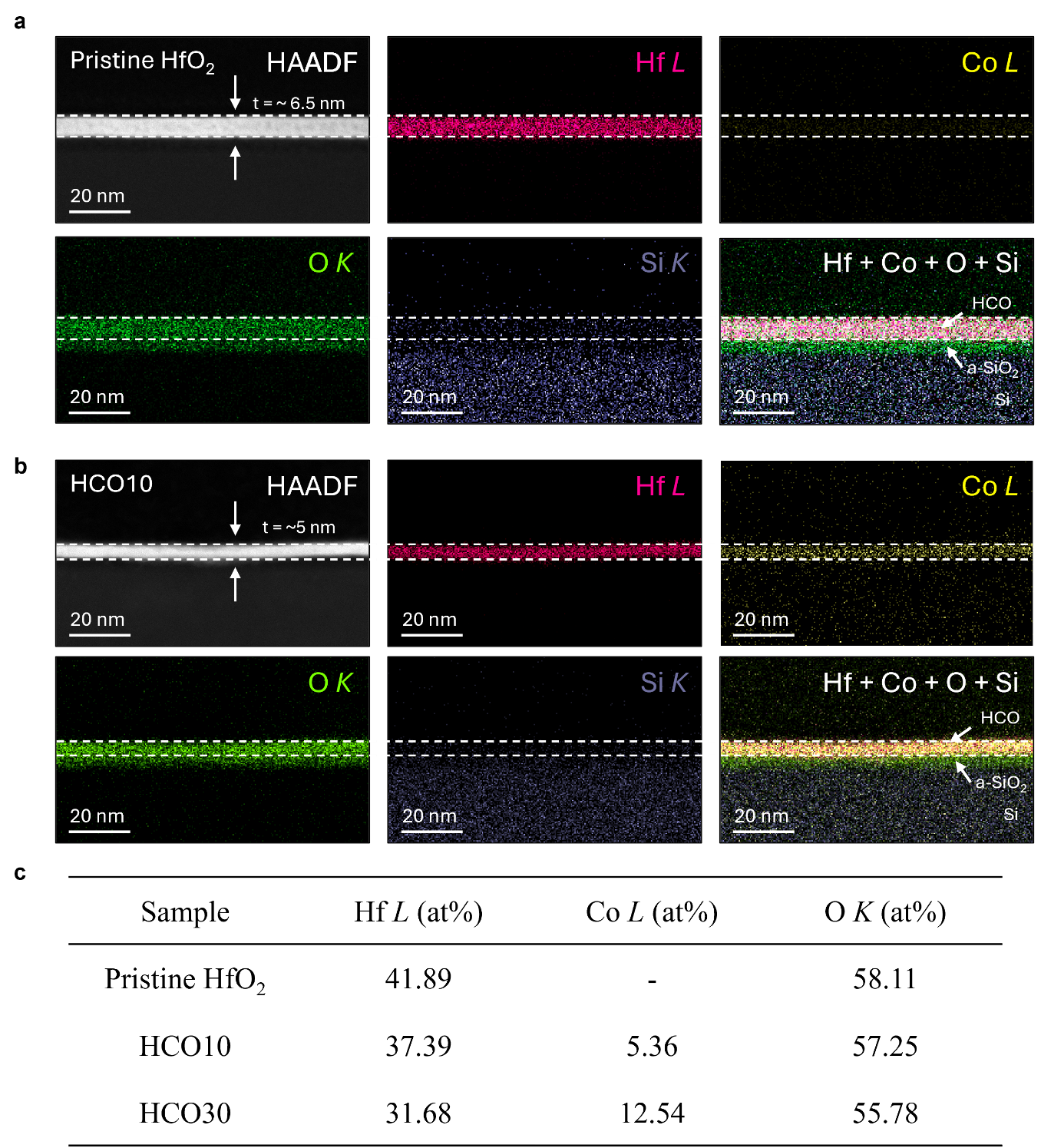


**Figure S4.** EDX elemental maps of Hf *L* (7.898 keV, pink), Co *L* (6.924 keV, yellow), O *K* (0.525 keV, green), and Si *K* (1.739 keV, purple) peaks for a) the pristine and b) HCO10 samples. c) Elemental composition of the pristine HfO_2_ and HCO films by standardless atomic ratio method.


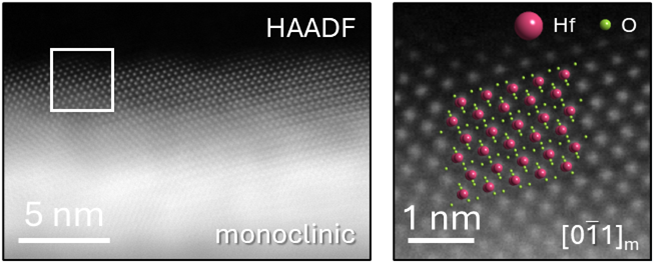


**Figure S5.** Representative high-resolution STEM images of monoclinic structure observed in HCO10 samples. Multiple STEM observations indicated that the pristine HfO_2_ has a monoclinic structure. As one of the results, the observed atomic structure corresponds to the atomic model of [0$\bar{1}$1]-oriented monoclinic HfO_2_.


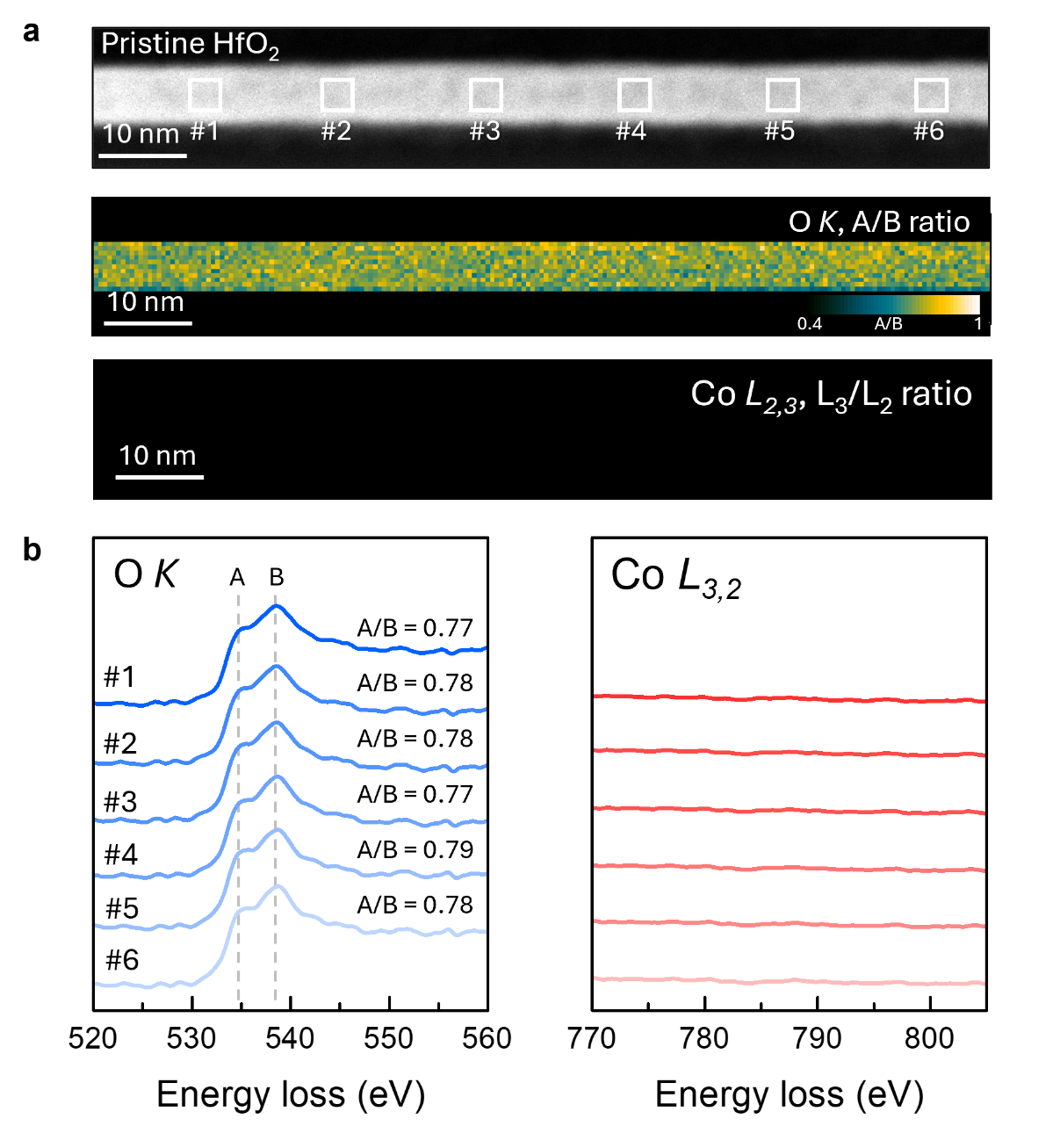


**Figure S6.** a) Maps of (top) A/B ratio in O *K* edge and (bottom) *L*_3_/*L*_2_ ratio in Co *L*_2,3_ edge for the pristine HfO_2_ film. b) Several O *K* and Co *L*_2,3_ EELS profiles extracted from five different regions randomly chosen for the pristine HfO_2_ film.


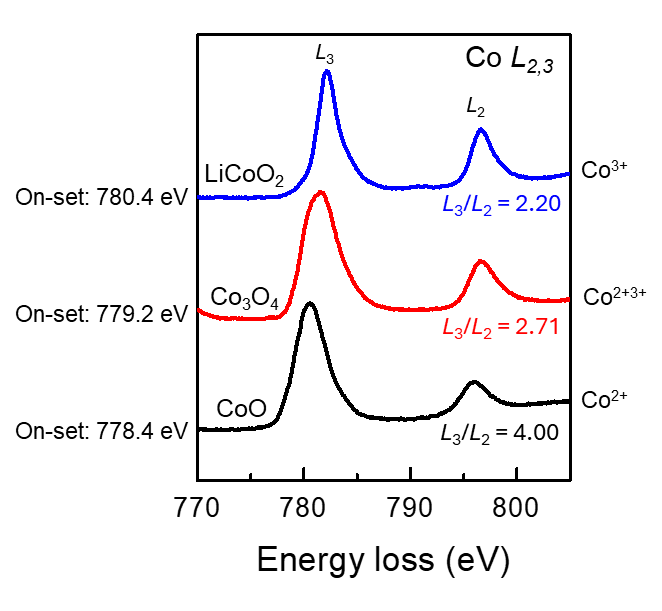


**Figure S7.** The estimated *L*_3_/*L*_2_ ratios and the onset energies of Co *L*_2,3_ edges obtained for standard samples: (blue) LiCoO_2_, (red) Co_3_O_4_, and (black) CoO, respectively.


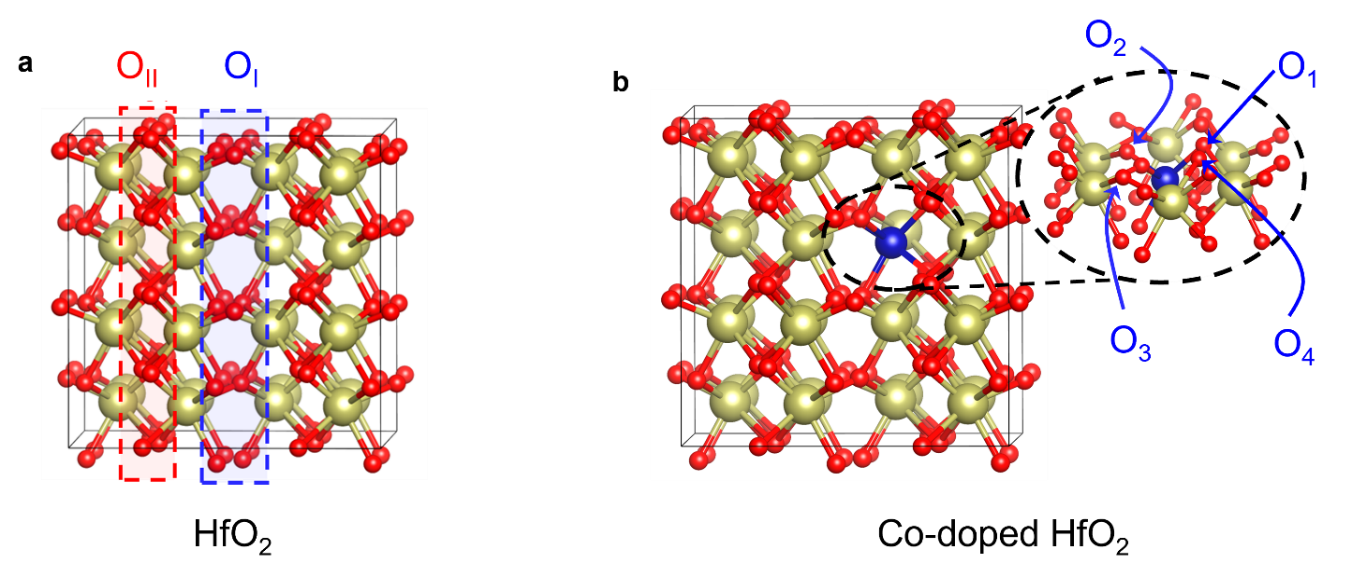


**Figure S8**. (a) Possible oxygen vacancy sites in *m*-HfO_2_ and *o*-HfO_2_. Here, O_I_ and O_II_ atoms have three and four nearest-neighboring Hf cations, respectively. (b) Possible oxygen vacancy sites in *m*-HCO and *o*-HCO. O_1_ atoms have three nearest-neighboring Hf cations and one Co cation. O_2_ atoms have two nearest-neighboring Hf cations and one Co cations. O_3_ and O_4_ atoms have three and four nearest-neighboring Hf cations, respectively.


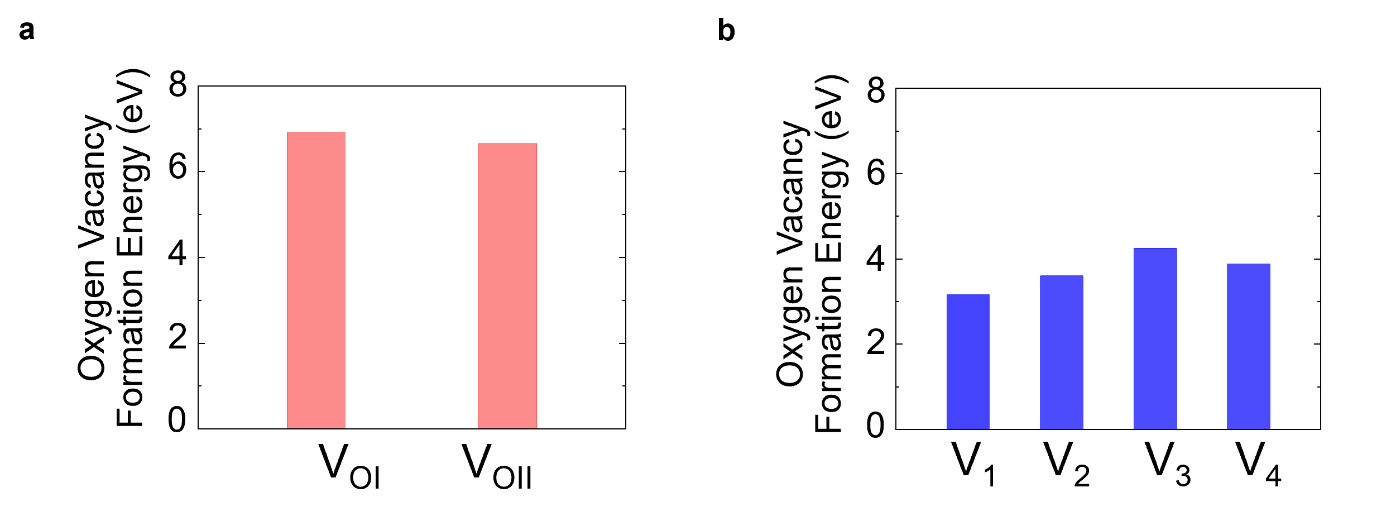


**Figure S9**. (a) Oxygen vacancy formation energies in (a) *o*-HfO_2_ (b) *o*-HCO.

**Figure S10.** Performance of HCO electrodes in non-enzymatic glucose sensing in 0.01 M PBS


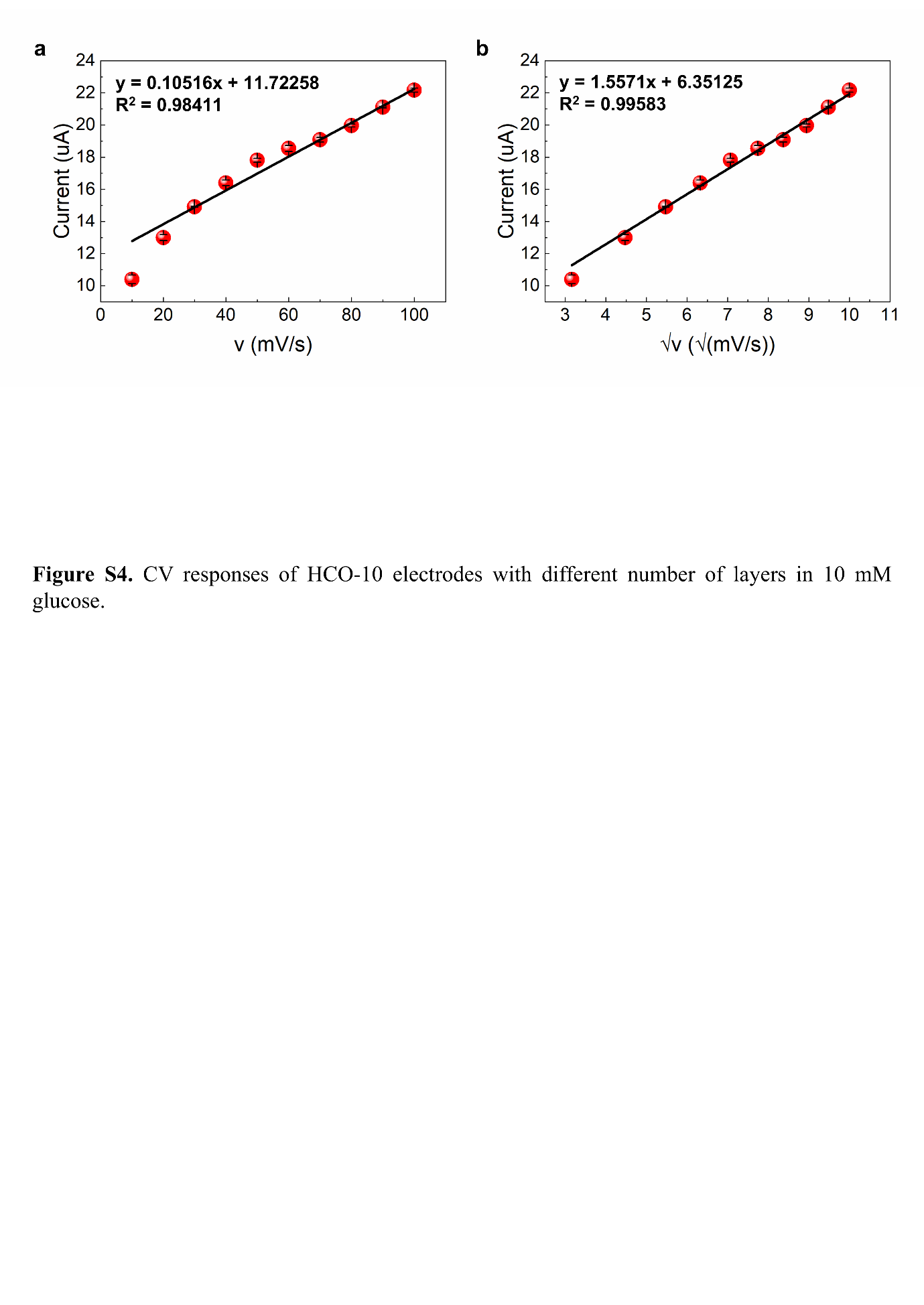


**Figure S11.** Corresponding calibration plots of peak current vs. the a) scan rate and b) square root of scan rate.


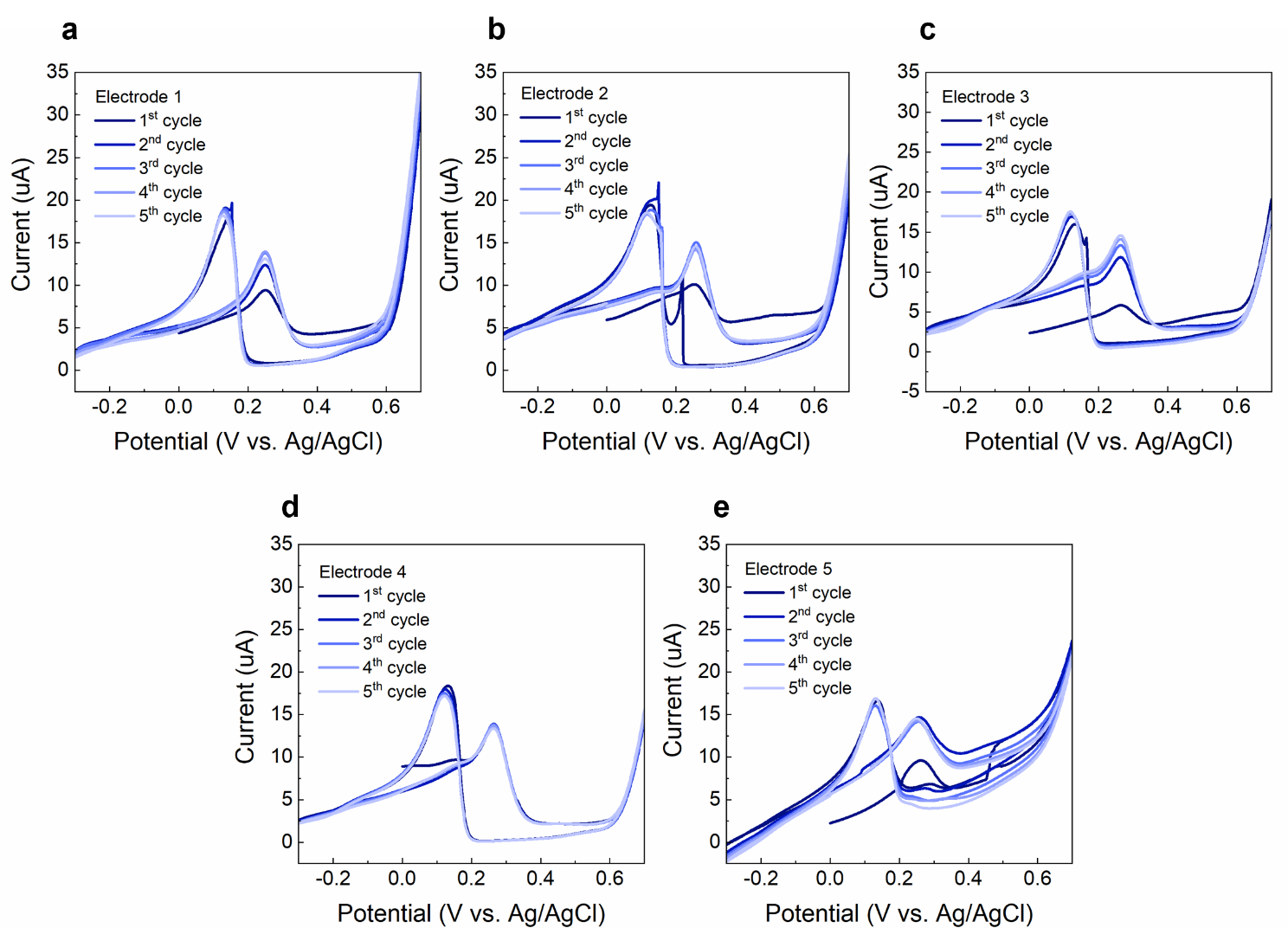


**Figure S12.** CV responses of five different electrodes coated with HCO for glucose sensing demonstrate the reproducibility of the sensing behavior.


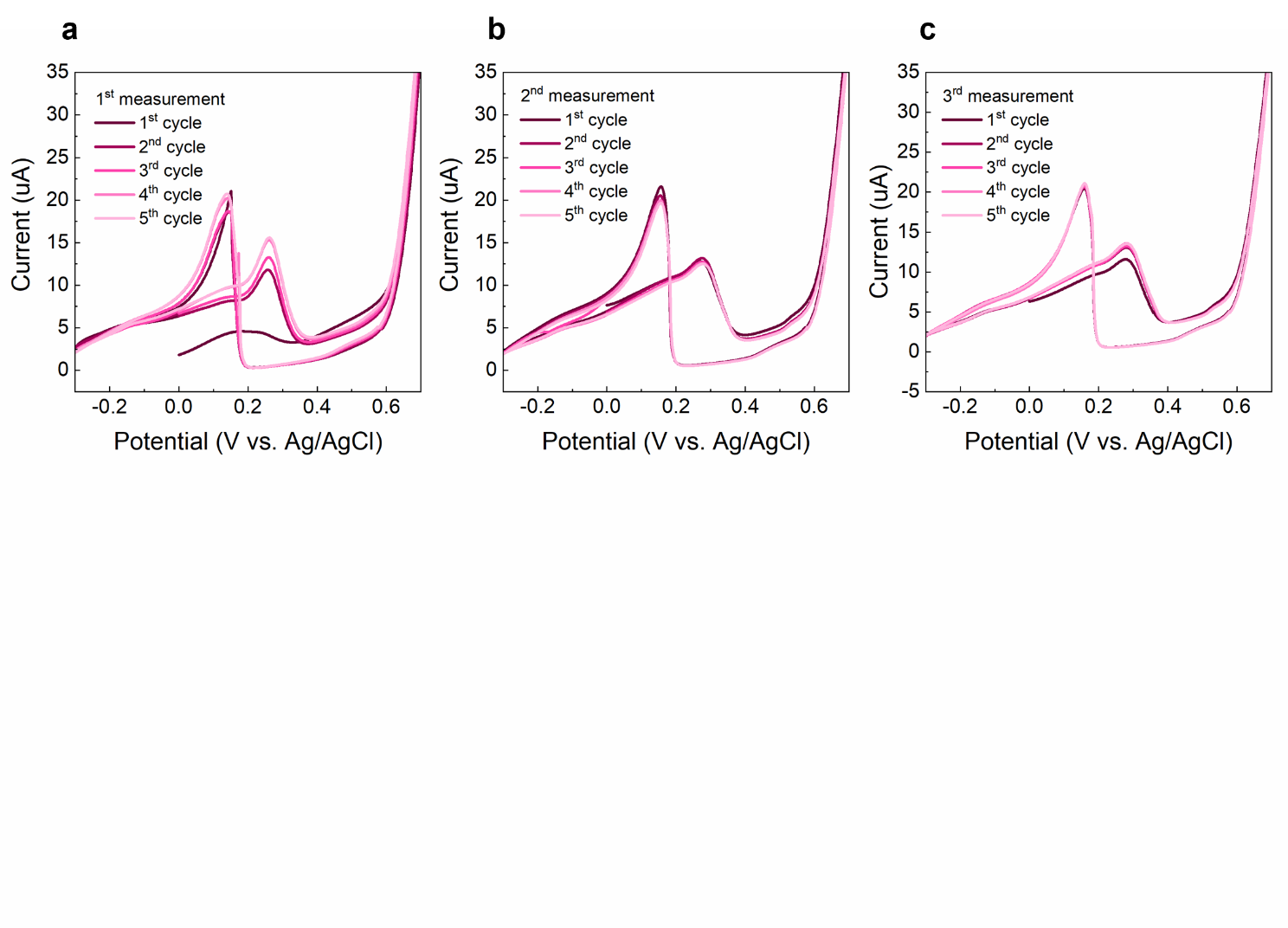


**Figure S13.** CV responses from three consecutive measurements validate the reproducibility of the glucose sensing behavior exhibited by the HCO electrode.


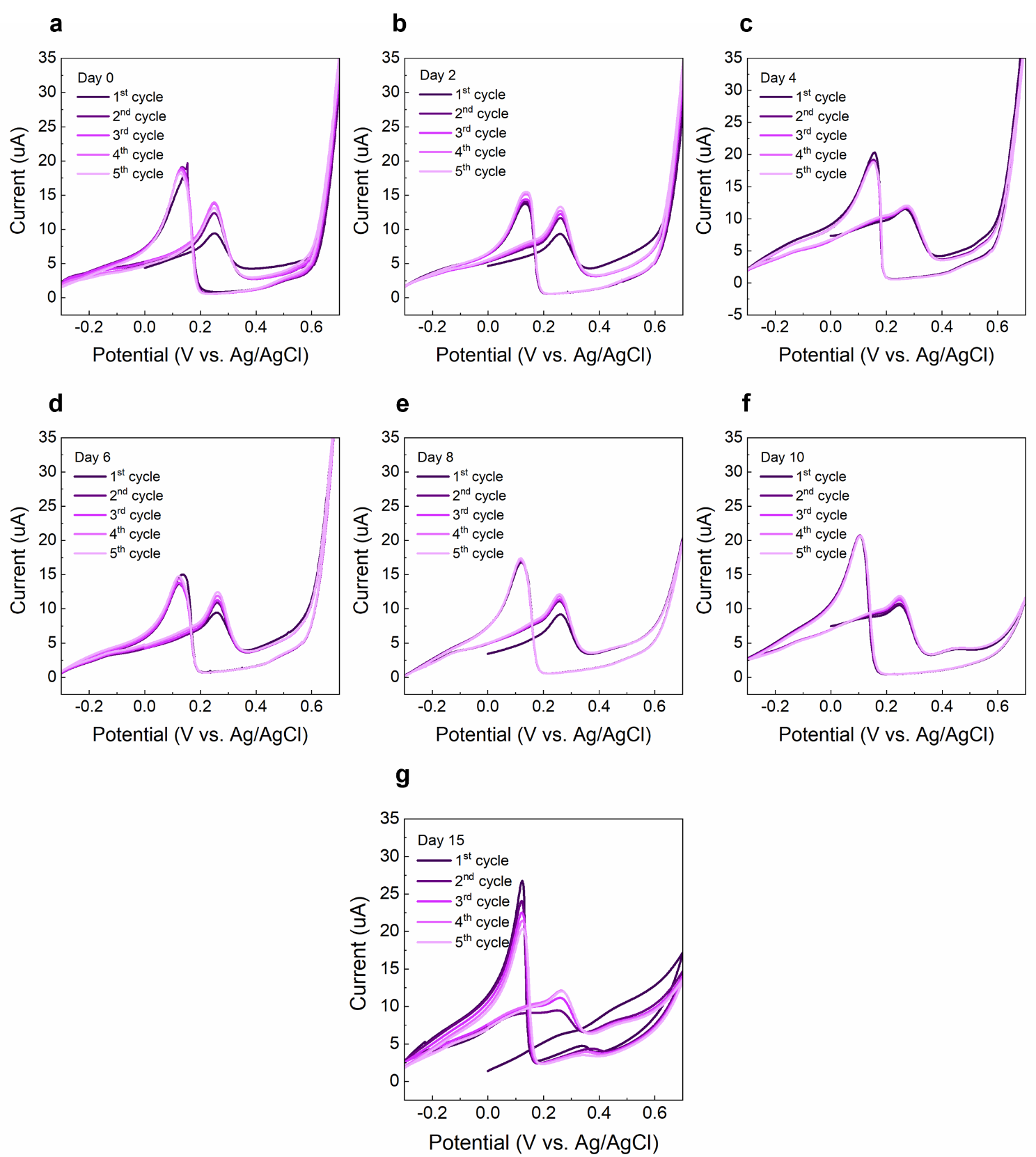


**Figure S14.** CV responses for stability testing each day (from day 0 to day 15) demonstrate the durability of the proposed HCO electrode.
